# Wearable sensor devices can automatically identify the ON-OFF status of patients with Parkinson's disease through an interpretable machine learning model

**DOI:** 10.3389/fneur.2024.1387477

**Published:** 2024-05-01

**Authors:** Xiaolong Wu, Lin Ma, Penghu Wei, Yongzhi Shan, Piu Chan, Kailiang Wang, Guoguang Zhao

**Affiliations:** ^1^Department of Neurosurgery, Xuanwu Hospital of Capital Medical University, Beijing, China; ^2^International Neuroscience Institute (China-INI), Beijing, China; ^3^Department of Neurorehabilitation, Rehabilitation Medicine of Capital Medical University, China Rehabilitation Research Centre, Beijing, China; ^4^Department of Neurology and Neurobiology, Xuanwu Hospital of Capital Medical University, Beijing, China; ^5^Beijing Municipal Geriatric Medical Research Center, Beijing, China

**Keywords:** Parkinson's disease, wearable sensor device, motor feature, interpretable machine learning model, MDS-UPDRS-Part III

## Abstract

**Introduction:**

Accurately and objectively quantifying the clinical features of Parkinson's disease (PD) is crucial for assisting in diagnosis and guiding the formulation of treatment plans. Therefore, based on the data on multi-site motor features, this study aimed to develop an interpretable machine learning (ML) model for classifying the “OFF” and “ON” status of patients with PD, as well as to explore the motor features that are most associated with changes in clinical symptoms.

**Methods:**

We employed a support vector machine with a recursive feature elimination (SVM-RFE) algorithm to select promising motion features. Subsequently, 12 ML models were constructed based on these features, and we identified the model with the best classification performance. Then, we used the SHapley Additive exPlanations (SHAP) and the Local Interpretable Model agnostic Explanations (LIME) methods to explain the model and rank the importance of those motor features.

**Results:**

A total of 96 patients were finally included in this study. The naive Bayes (NB) model had the highest classification performance (AUC = 0.956; sensitivity = 0.8947, 95% CI 0.6686–0.9870; accuracy = 0.8421, 95% CI 0.6875–0.9398). Based on the NB model, we analyzed the importance of eight motor features toward the classification results using the SHAP algorithm. The Gait: range of motion (RoM) Shank left (L) (degrees) [Mean] might be the most important motor feature for all classification horizons.

**Conclusion:**

The symptoms of PD could be objectively quantified. By utilizing suitable motor features to construct ML models, it became possible to intelligently identify whether patients with PD were in the “ON” or “OFF” status. The variations in these motor features were significantly correlated with improvement rates in patients' quality of life. In the future, they might act as objective digital biomarkers to elucidate the changes in symptoms observed in patients with PD and might be used to assist in the diagnosis and treatment of patients with PD.

## Introduction

Parkinson's disease (PD) is a chronic degenerative disease of the central nervous system and is characterized by the degeneration or loss of dopaminergic neurons in the substantia nigra and the appearance of Lewy bodies. The clinical features of PD include bradykinesia, rest tremor, muscular rigidity, and postural impairment (1–3). As a result, the objective quantitative assessment of these clinical features plays a crucial role in diagnosis and guiding the formulation of treatment plans. The Movement Disorder Society-Unified Parkinson's Disease Rating Scale (MDS-UPDRS) is commonly used to measure the severity of patients with PD (4). The MDS-UPDRS-Part III is widely used to assess movement disorder in patients with PD. It is a semi-quantitative measurement and consists of 18 items (4). However, some limitations might be found in the clinical assessment by the MDS-UPDRS-Part III. First, it is not an objective, quantifiable evaluation method, and the evaluation process requires a specific physician and a lot of time. Second, the evaluation results are affected by the doctor's experience, the cognitive performance of the patients, and the surrounding environment; thus, the accuracy and objectivity of the evaluation results are limited (5–7). Finally, the symptoms of patients with PD fluctuate significantly, which poses great challenges for clinical assessment (8).

In recent years, the use of different sensor devices for the quantitative evaluation of motor ability in patients with PD has been increasingly explored. Many studies supported the reliability of motor data collected by wearable sensor devices (WSD) to assist in the diagnosis of patients with PD and the assessment of disease progression and to guide clinical practice (9–14). Some studies have combined machine learning (ML) or deep learning (DL) algorithms with WSD to estimate MDS-UPDRS-Part III and assist in the diagnosis of PD (15–17). Although it brought great opportunities and potential for an intelligent evaluation of PD, some challenges might affect the accuracy of their results (15, 16, 18). First, there is still no consensus on what motor features are most relevant to the changes in clinical symptoms (between the “OFF” and “ON” status, or between patients with PD and healthy elderly people). In addition, some studies had small sample sizes, and the WSDs were placed only on a single site to collect patient motor features. Moreover, some WSDs were cumbersome and inconvenient to wear, causing inconvenience to patients with PD during use. Finally, although the classification performance of multiple predictive models proved to be promising, their interpretability for classifying the “OFF” and “ON” status of patients with PD is still limited (19). These factors hinder the application of WSDs in objectively quantifying the clinical features of patients with PD. The question is whether we can maximize the accurate quantification of clinical features in patients with PD using simpler wearing methods and fewer motor features.

Therefore, based on the data on multi-site motor features, this study aimed to develop an interpretable ML model for classifying the “OFF” and “ON” status of patients with PD, as well as to explore the motor features most associated with changes in clinical symptoms. In addition, we used SHapley Additive exPlanations (SHAP) and Local Interpretable Model agnostic Explanations (LIME) methods (20, 21) to explain the model and rank the importance of those motor features.

## Materials and methods

### Study design

This was a retrospective observational study. It was approved by the Institutional Ethics Committee of Xuanwu Hospital and performed according to the principles of the Declaration of Helsinki. All personal information was made anonymous before analysis. We retrospectively analyzed the following clinical information: personal and medical history, the history of drug substance intake, current medication usage, MDS-UPDRS-Part III (4), the results of motor assessment, Hoehn and Yahr scale (HY) (22), and Activities of Daily Living (ADL, assessed by the Barthel Index) (23). We applied the levodopa challenge test to assess the responsiveness of patients with PD to levodopa medication and to distinguish between patients in the “ON” and “OFF” status (24, 25). The “OFF” status was defined as being off dopamine agonists for 72 h and off antiparkinsonian drugs for 12 h. The “ON” status was the best statement after taking antiparkinsonian medications (~2 h after taking the medicine). The MDS-UPDRS-Part III scores and motor assessment were evaluated at 8:00 AM during the “OFF” status. After taking the medicine, MDS-UPDRS-Part III was recorded at 1, 2, 3, and 4 h, and the best performance was selected for statistical analyses. MDS-UPDRS-Part III improvement rate = (MDS-UPDRS-Part III score “OFF” – MDS-UPDRS-Part III score “ON”)/MDS-UPDRS-Part III score “OFF” × 100%. ADL improvement rate = (ADL score in the “ON” status – ADL score in the “OFF” status)/ADL score in the “OFF” status × 100%.

### Patients

From January 2019 to December 2020, data on patients with PD who were recruited from the neurology clinic of Xuanwu Hospital, Beijing, China, were retrospectively collected. According to the Movement Disorder Society Clinical Diagnostic Criteria for PD (26), they were diagnosed with clinically established PD by a movement disorders specialist. The inclusion and exclusion criteria are shown in Supplementary Table S1.

### Tools for motor assessment

**Figure 1:**
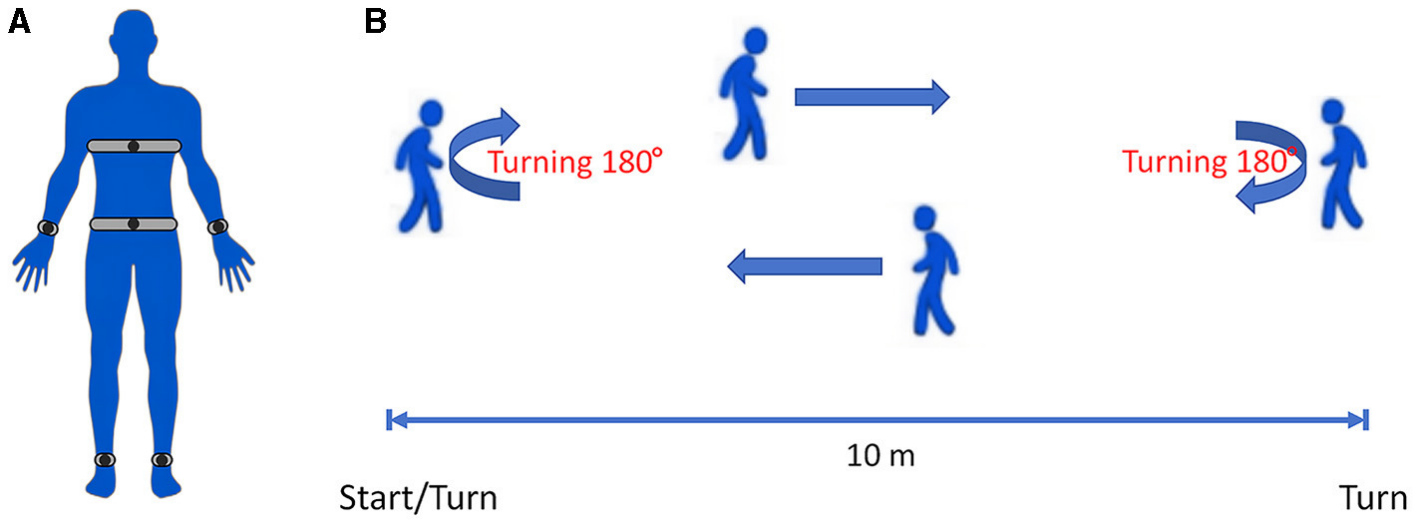
**(A)** Schematic diagram showing the positions of six Opal™ Movement Monitors on the patient: the bilateral wrists and ankles, the anterior sternum, and the lower back. **(B)** Schematic diagram of the IWalk Test. All patients were required to walk at least 1 min.

Six Opal™ Movement Monitors (APDM, Inc., Portland, OR, United States) were placed on the bilateral wrists and ankles, the anterior sternum, and the lower back (Figure 1A). Each movement monitor included a three-axis accelerometer, a three-axis gyro, a three-axis magnetometer, and a temperature sensor. It was secured to the patient using optional straps that could be connected to the host via wireless communication. For detailed information about Opal™ Movement Monitors and gait data, please visit the official website (APDM, Inc., Portland, OR, United States, https://apdm.com/wearable-sensors/).

### Motor assessment procedures

We utilized the Instrumented Long Walk (IWalk) Test protocol for gait data collection. The IWalk analysis algorithms automatically process recorded movement data and provide objective measures related to gait and turning. For more detailed information, please visit the official website (APDM, Inc., Portland, OR, United States, https://apdm.com/wearable-sensors/). The patients were asked to wear comfortable clothing and walking shoes that did not bind or impede their movement in any significant way. After signing the informed consent form, they wore six Opal™ Movement Monitors and walked on a straight 10 m walkway at a comfortable pace. All patients walked from the start point to the end of the 10 m walkway and then turned around to return to the starting point (Figure 1B). All patients were required to walk for at least 1 min. These recorded parameters could be transmitted to the computing center in real time by wireless transmission technology for three-dimensional movement posture reconstruction, and then the gait, posture balance, arm swing, movement coordination, etc. could be evaluated.

### Statistical analysis

The R software (version 4.1.3; R Core Team) was used to perform all statistical analyses. If there were missing values in the data included in this study, the k-nearest neighbors interpolation method was used for imputation (27). Continuous quantitative variables are expressed as means, standard deviations, medians, and interquartile ranges (IQRs). Categorical variables are expressed as total numbers and percentages. The support vector machine – recursive feature elimination (SVM-RFE) algorithm was used to filter the motor features with a five-fold cross validation and five re-samplings (28). We used the Wilcoxon signed-rank test to compare the differences in motor features between the “OFF” and “ON” status. The correlation between the ADL improvement rate and the improvement rate of motor features was explored using the Pearson correlation test.

### Classification model construction

A total of 12 ML models, namely, Adaboost, LogitBoost, XGBoost, logistic regression (LR), random forest (RF), support vector machine (SVM), neural network (NN), k-nearest neighbors (KNN), decision tree C5.0, naive Bayes (NB), gradient boosting machine (GBM), and multilayer perceptron (MLP), were used to develop the classification models. The 10-fold cross-validation and RandomSearch for hyperparameters were used for training each ML model. We calculated the area under the receiver operating characteristic curve, sensitivity, specificity, accuracy, positive predictive values, negative predictive values, recall, and F1 score. The area under the curve (AUC) was used as the main index to evaluate the classification performance of each model. If the AUC value was the same or similar, the sensitivity, specificity, accuracy, positive predictive values, negative predictive values, recall, and F1 score were referred.

### Interpretation tool for the model

The interpretation of the ML model of the best classification performance was performed using the SHAP and LIME methods (20, 21). SHAP is a common method to analyze the contribution and influence of each motor feature toward the overall performance of the classification model. The SHAP value is calculated to show how important each motor feature is for the target variable, either positively or negatively. The SHAP and LIME methods were used to explore the contributions of each variable to the overall performance of the classification model and the classification of an instance, respectively.

## Results

### Patient characteristics

From January 2019 to December 2020, a total of 110 patients with PD were screened, and based on the inclusion and exclusion criteria, 96 patients were ultimately included in this study. The reasons for excluding 14 patients were as follows: some patients were unable to complete the IWalk Test task owing to heart and orthopedic diseases; some patients had other neurological or psychiatric disorders; some had concurrent other neurological or psychiatric disorders; and a significant amount of information was missing for some patients. Supplementary Figure S1 presents the patient screening process. The demographic and clinical information of patients with PD are presented in Table 1. We randomly divided a total of 192 patients (the “OFF” and “ON” status of 96 patients) into two parts: 80% (training dataset, *n* = 154) of the subjects were used to train the classification model, and 20% (validating dataset, *n* = 38) were used to validate. Then, based on the training dataset, we used the SVM-RFE algorithm to find eight potential motor features for developing the classification model.

**Table 1:** Demographic and clinical information of patients with PD.

**Characteristics**	**Baseline**
Demographic	
Patient numbers	96
Age, Median [IQR]	62 [10.25]
Gender, male [%]	41 [42.71%]
Education, high school or higher [%]	57 [59.38%]
Clinical	
Disease duration, Median [IQR]	9 [6]
HY stage (the “OFF” status)	
I/II/III/IV/V	0/53/35/6/2
MDS-UPDRS-III (the “OFF” status), Median [IQR]	57 [26]
MDS-UPDRS-III (the “ON” status), Median [IQR]	30.5 [20]
MDS-UPDRS-III improvement rate, Median [IQR]	47.3% [26.19%]
LEDD (mg), Median [IQR]	300 [75]

LEDD, levodopa-equivalent daily dose; SD, standard deviation; IQR, interquartile range; HY stage, Hoehn & Yahr stage.

### Machine learning model construction and evaluation

**Figure 2:**
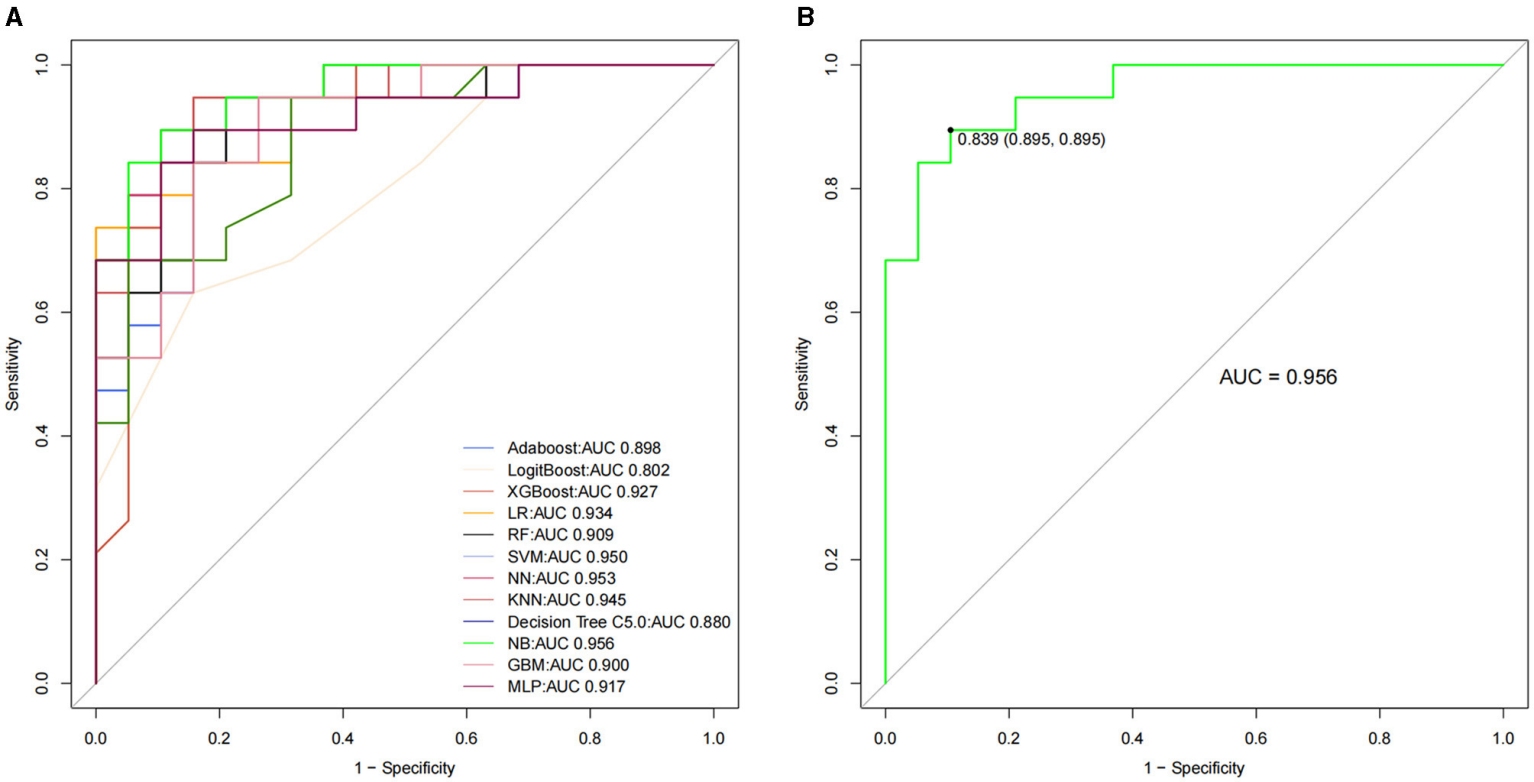
The receiver operating characteristic curve among the 12 classification models for patients with PD **(A)**. The NB model had the highest classification performance **(B)**. PD = Parkinson's disease, NB = naive Bayes.

Based on the training dataset, Adaboost, LogitBoost, XGBoost, LR, RF, SVM, NN, KNN, decision tree C5.0, NB, GBM, and MLP classification models were constructed, and the AUCs of the validating datasets were 0.898, 0.802, 0.927, 0.934, 0.909, 0.95, 0.953, 0.945, 0.88, 0.956, 0.9, and 0.917, respectively (Table 2 and Figure 2). After a comprehensive comparison, the NB model had the highest classification performance (AUC = 0.956; sensitivity = 0.8947, 95% CI 0.6686–0.9870; accuracy = 0.8421, 95% CI 0.6875–0.9398; positive predictive values = 0.8095, 95% CI 0.5809–0.9455; negative predictive values = 0.8824, 95% CI 0.6356–0.9854; recall = 0.8947; F1 score = 0.85).

**Table 2:** Performance of each classification model in the validating dataset.

**Model**	**AUC**	**Sensitivity**	**Accuracy**	**Positive Pred Value**	**Negative Pred Value**	**Recall**	**F1 score**
Adaboost	0.898	0.7895 (0.5443, 0.9395)	0.8158 (0.6567, 0.9226)	0.8333 (0.5858, 0.9642)	0.8000 (0.5634, 0.9427)	0.6842	0.7428
LogitBoost	0.802	0.6842 (0.4345, 0.8742)	0.6842 (0.5135, 0.8250)	0.6842 (0.4345, 0.8742)	0.6842 (0.4345, 0.8742)	0.6842	0.6842
XGBoost	0.927	0.7895 (0.5443, 0.9395)	0.8421 (0.6875, 0.9398)	0.8824 (0.6356, 0.9854)	0.8095 (0.5809, 0.9455)	0.7894	0.8333
LR	0.934	0.7895 (0.5443, 0.9395)	0.8421 (0.6875, 0.9398)	0.8824 (0.6356, 0.9854)	0.8095 (0.5809, 0.9455)	0.7894	0.8333
RF	0.909	0.7895 (0.5443, 0.9395)	0.8158 (0.6567, 0.9226)	0.8333 (0.5858, 0.9642)	0.8000 (0.5634, 0.9427)	0.7894	0.8108
SVM	0.95	0.7895 (0.5443, 0.9395)	0.8421 (0.6875, 0.9398)	0.8824 (0.6356, 0.9854)	0.8095 (0.5809, 0.9455)	0.7894	0.8333
NN	0.953	0.7895 (0.5443, 0.9395)	0.8421 (0.6875, 0.9398)	0.8824 (0.6356, 0.9854)	0.8095 (0.5809, 0.9455)	0.7894	0.8333
KNN	0.945	0.8421 (0.6042, 0.9662)	0.8684 (0.7191, 0.9559)	0.8889 (0.6529, 0.9862)	0.8500 (0.6211, 0.9679)	0.8421	0.8648
DT C5.0	0.88	0.7895 (0.5443, 0.9395)	0.7368 (0.5690, 0.8660)	0.7143 (0.4782, 0.8872)	0.7647 (0.5010, 0.9319)	0.7894	0.75
NB	0.956	0.8947 (0.6686, 0.9870)	0.8421 (0.6875, 0.9398)	0.8095 (0.5809, 0.9455)	0.8824 (0.6356, 0.9854)	0.8947	0.85
GBM	0.9	0.7895 (0.5443, 0.9395)	0.8158 (0.6567, 0.9226)	0.8333 (0.5858, 0.9642)	0.8000 (0.5634, 0.9427)	0.7894	0.8108
MLP	0.917	0.7895 (0.5443, 0.9395)	0.8421 (0.6875, 0.9398)	0.8824 (0.6356, 0.9854)	0.8095 (0.5809, 0.9455)	0.7894	0.8333

AUC, area under the curve; LR, logistic regression; RF, random forest; SVM, support vector machine; NN, neural network; KNN, k-nearest neighbors; DT, decision tree; NB, naive Bayes; GBM, gradient boosting machine; MLP, multilayer perceptron.

### Interpretation of the NB model with the SHAP and LIME methods

**Figure 3:**
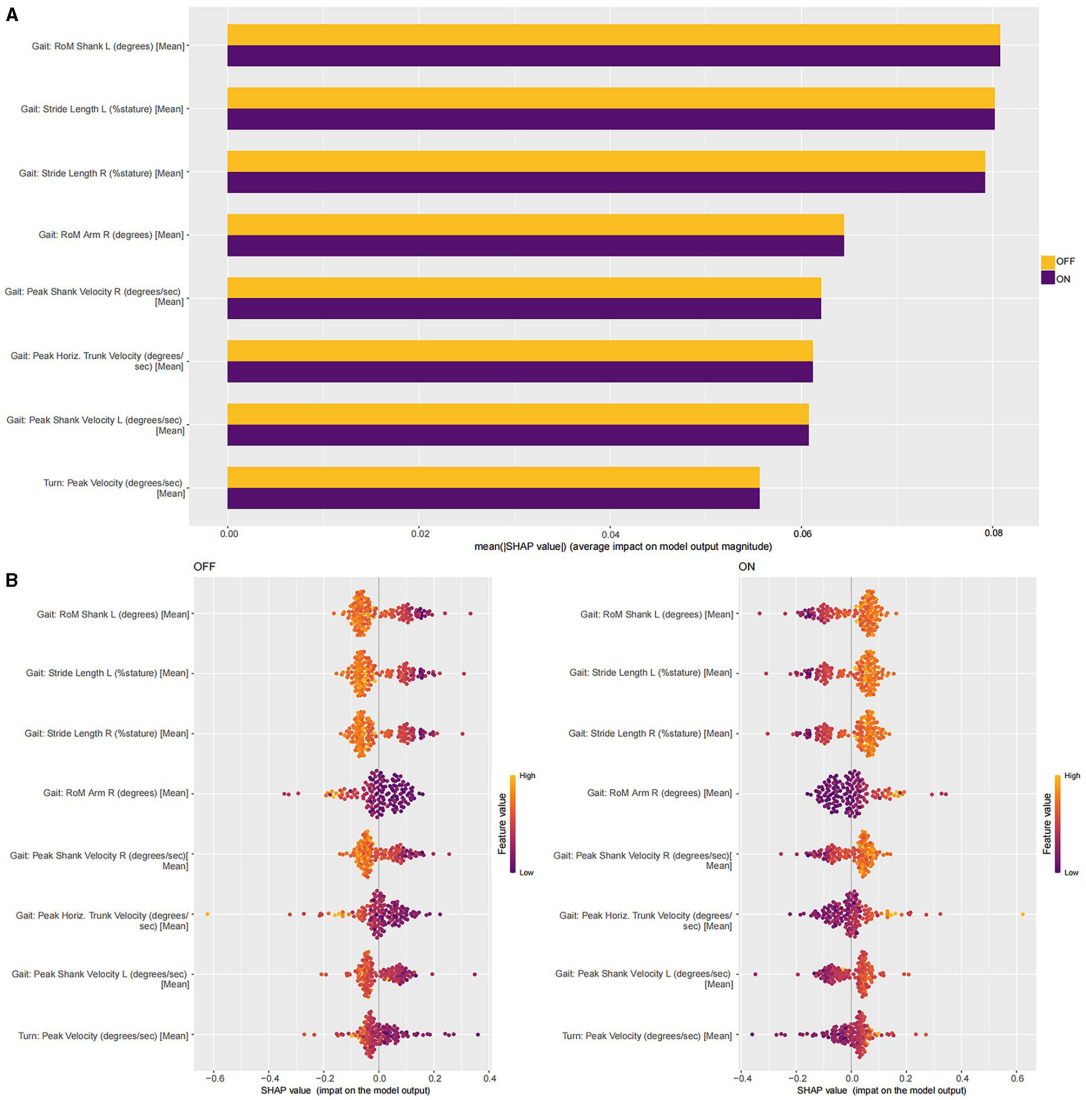
**(A)** The SHAP interpretability for the contributions of each motor feature. **(B)** The SHAP values of all motor features in the NB model in the training dataset.

Based on the NB model, we analyzed the importance of eight motor features toward the classification results using the SHAP algorithm. The motor feature importance ranking is shown in Figure 3A. The Gait: range of motion (RoM) Shank left (L) (degrees) [Mean] might be the most important motor feature for all classification horizons, followed by the Gait: Stride Length L (%stature) [Mean], the Gait: Stride Length R (%stature) [Mean], the Gait: RoM Arm R (degrees) [Mean], the Gait: Peak Shank Velocity R (degrees/s) [Mean], the Gait: Peak Horiz. Trunk Velocity (degrees/s) [Mean], the Gait: Peak Shank Velocity L (degrees/s) [Mean], and the Turn: Peak Velocity (degrees/s) [Mean]. There were positive and negative correlations between the motor features and classification results. In Figure 3B, the colors of points show whether the motor feature was high (in aurantium) or low (in purple) in this study. We found that the increase in the value of all motor features had a positive effect on the “ON” status, driving the prediction of the “ON” status.

**Figure 4:**
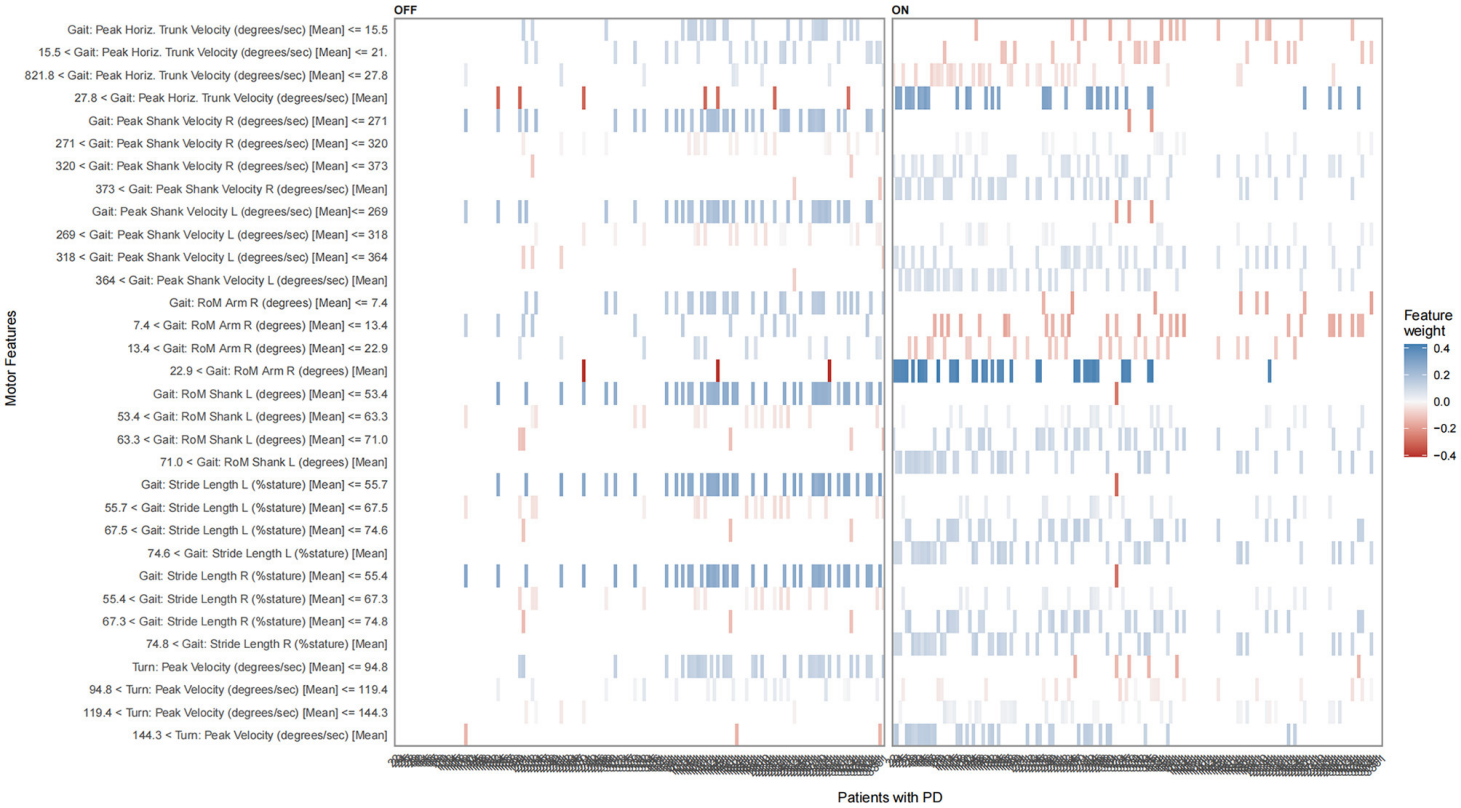
The LIME plot of the NB model in the training datasets. The blue bar represents a positive effect, and the darker and denser the blue bar, the more likely it is to have a positive effect. The red bars represent negative effects, and the darker and denser the red, the more likely they are to have negative effects.

The LIME method was also used to explain how eight motor features contributed to the classification results (Figure 4). The sequentially important motor features that contributed to the “OFF” status included the Gait: RoM Shank L (degrees) [Mean] < = 53.4, the Gait: Stride Length L (%stature) [Mean] < = 55.7, the Gait: Stride Length R (%stature) [Mean] < = 55.4, the Turn: Peak Velocity (degrees/s) [Mean] < = 94.8, the Gait: RoM Arm R (degrees) [Mean] < = 7.4, the 7.4 < Gait: RoM Arm R (degrees) [Mean] < = 13.4, the 13.4 < Gait: RoM Arm R (degrees) [Mean] < = 22.9, the Gait: Peak Shank Velocity L (degrees/s) [Mean] < = 269, the Gait: Peak Shank Velocity R (degrees/s) [Mean] < = 271, the Gait: Peak Horiz. Trunk Velocity (degrees/s) [Mean] < = 15.5, and the 15.5 < Gait: Peak Horiz. Trunk Velocity (degrees/s) [Mean] < = 21.8. The sequentially important motor features that contributed to the “ON” status included the 71.0 < Gait: RoM Shank L (degrees) [Mean], the 63.3 < Gait: RoM Shank L (degrees) [Mean] < = 71.0, the 74.6 < Gait: Stride Length L (%stature) [Mean], the 67.5 < Gait: Stride Length L (%stature) [Mean] < = 74.6, the 74.8 < Gait: Stride Length R (%stature) [Mean], the 67.3 < Gait: Stride Length R (%stature) [Mean] < = 74.8, the 144.3 < Turn: Peak Velocity (degrees/s) [Mean], the 22.9 < Gait: RoM Arm R (degrees) [Mean], the 364 < Gait: Peak Shank Velocity L (degrees/s) [Mean], the 318 < Gait: Peak Shank Velocity L (degrees/s) [Mean] < = 364, the 373 < Gait: Peak Shank Velocity R (degrees/s) [Mean], the 320 < Gait: Peak Shank Velocity R (degrees/s) [Mean] < = 373, and the 27.8 < Gait: Peak Horiz. Trunk Velocity (degrees/s) [Mean]. Similar to the SHAP method, we also found that the increase in the value of all motor features has a positive effect on the “ON” status, driving the prediction of the “ON” status.

### Differences of eight motor feature values between the “OFF” and “ON” status and correlation analysis

**Figure 5:**
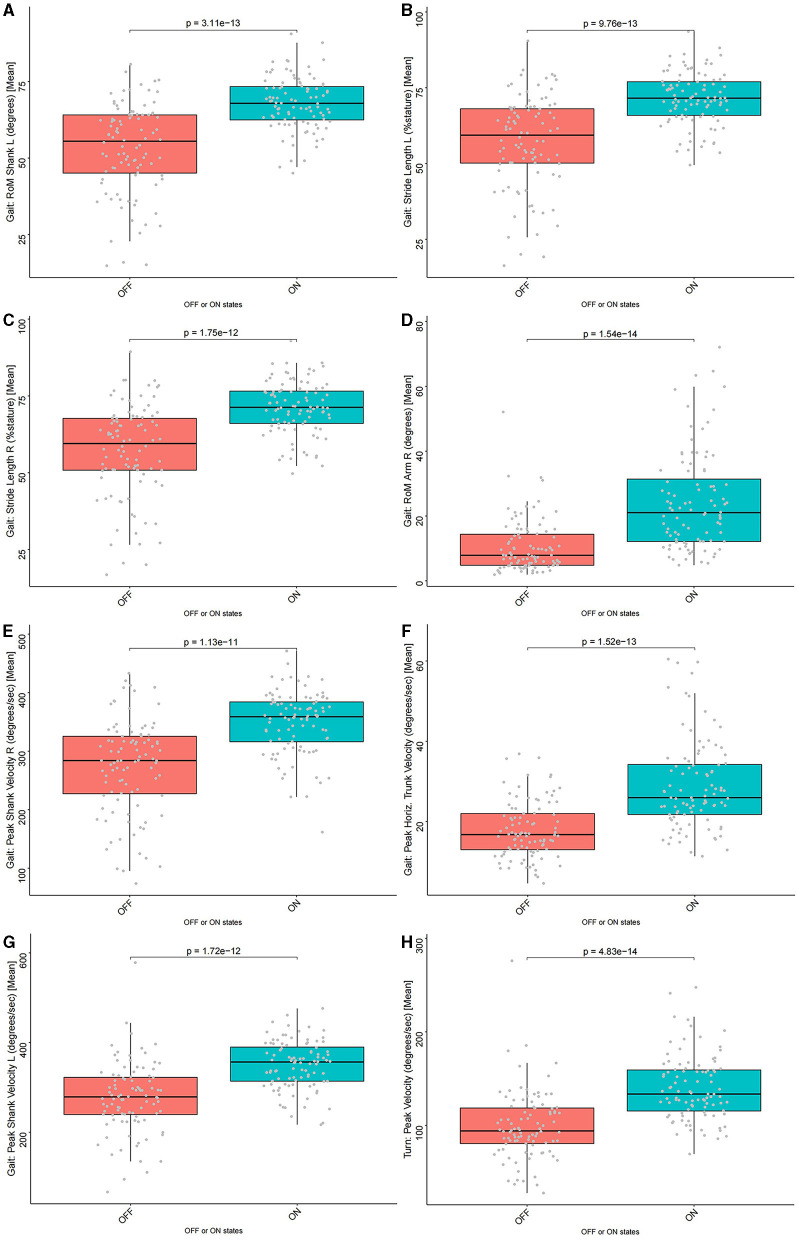
The comparison of eight motor feature values between the “OFF” and “ON” status. All motor features had significantly higher values in the “ON” status than in the “OFF” status (*p* < 0.05). **(A)** Gait: RoM Shank L (degrees) [Mean] (*p* = 3.11e-13), **(B)** Gait: Stride Length L (%stature) [Mean] (*p* = 9.76e-13), **(C)** Gait: Stride Length R (%stature) [Mean] (*p* = 1.75e-12), **(D)** Gait: RoM Arm R (degrees) [Mean] (*p* = 1.54e-14), **(E)** Gait: Peak Shank Velocity R (degrees/s) [Mean] (*p* = 1.13e-11), **(F)** Gait: Peak Horiz. Trunk Velocity (degrees/s) [Mean] (*p* = 1.52e-13), **(G)** Gait: Peak Shank Velocity L (degrees/s) [Mean] (*p* = 1.72e-12), and **(H)** Turn: Peak Velocity (degrees/s) [Mean] (*p* = 4.83e-14).

**Figure 6:**
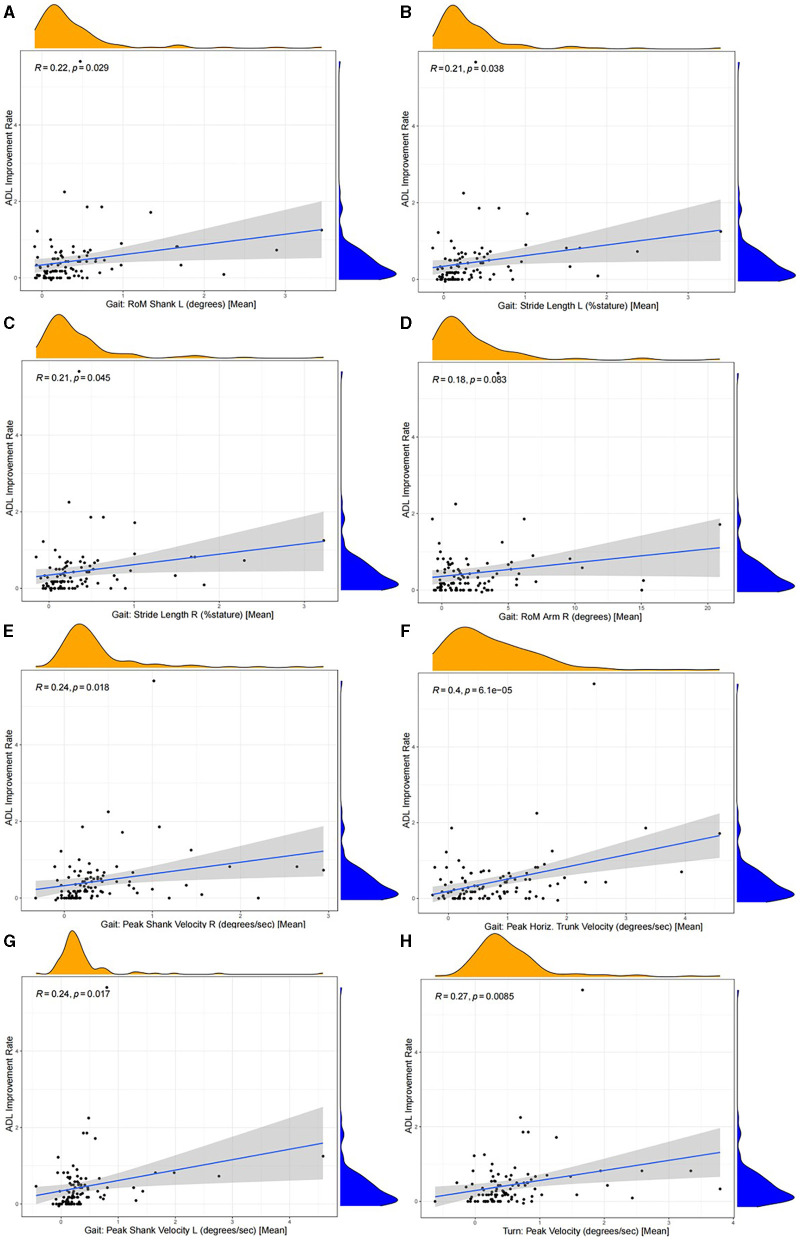
The correlation (Pearson correlation analysis) between the improvement rate of each motor feature and the ADL improvement rate. The improvement rate of each motor feature was significantly correlated positively with the ADL improvement rate of patients (*p* < 0.05), except for Gait: RoM Arm R (degrees) [Mean] (R = 0.18, *p* = 0.083). **(A)** Gait: RoM Shank L (degrees) [Mean] (R = 0.22, *p* = 0.029), **(B)** Gait: Stride Length L (%stature) [Mean] (R = 0.21, *p* = 0.038), **(C)** Gait: Stride Length R (%stature) [Mean] (R = 0.21, *p* = 0.045), **(D)** Gait: RoM Arm R (degrees) [Mean] (R = 0.18, *p* = 0.083), **(E)** Gait: Peak Shank Velocity R (degrees/s) [Mean] (R = 0.24, *p* = 0.018), **(F)** Gait: Peak Horiz. Trunk Velocity (degrees/s) [Mean] (R = 0.4, *p* = 6.1e−05), **(G)** Gait: Peak Shank Velocity L (degrees/s) [Mean] (R = 0.24, *p* = 0.017), and **(H)** Turn: Peak Velocity (degrees/s) [Mean] (R = 0.27, *p* = 0.0085).

The Wilcoxon signed-rank test was performed to compare the values of eight motor features in the “OFF” and “ON” status, and the results showed that the motor features had significantly higher values in the “ON” status than in the “OFF” status (*p* < 0.05). The details are available in Figure 5 and Supplementary Table S2. The Pearson correlation analysis presented that the improvement rate of each motor feature was significantly and positively correlated with the ADL improvement rate of patients (*p* < 0.05), except for Gait: RoM Arm R (degrees) [Mean] (*p* = 0.083) (Figure 6). This indicated that, compared to the “OFF” status, in the “ON” status, the values of the eight motor features of patients with PD were significantly higher, thus providing patients with a better motor status and quality of life.

## Discussion

In our study, we aimed to develop an interpretable ML model for classifying the “OFF” and “ON” status of patients with PD, as well as to explore the motor features most associated with changes in clinical symptoms. In total, 12 ML classification models were developed and validated to classify the “OFF” and “ON” status of patients with PD. The classification performance of the XGBoost model outperformed the Adaboost, LogitBoost, LR, RF, SVM, NN, KNN, decision tree C5.0, NB, GBM, and MLP models. We performed the interpretation for the NB model by the SHAP and LIME methods, which provided a guarantee for its performance and clinical interpretability. In addition, it helped us better understand the classification process of the NB model. Some important motor features and the importance of ranking those features were identified to be associated with identifying the status of patients with PD; this was performed by the SHAP and LIME methods to interpret the NB model. A total of five motor features describing the lower extremities, two motor features describing the trunk of the body, and one motor feature describing the upper limb were included (Figure 3). Then it was confirmed that each of the motor features included in the NB model had significant differences between the “OFF” and “ON” status. The correlation analysis showed the ADL improvement rates were closely related to the improvement rates for each motor feature. The most important finding was that the motor features of the lower limbs might be better for classification performance and could more truly reflect the motor symptoms of patients, mainly including the Gait: RoM Shank L (degrees) [Mean], the Gait: Stride Length L (%stature) [Mean], the Gait: Stride Length R (%stature) [Mean], the Gait: Peak Shank Velocity R (degrees/s) [Mean], and the Gait: Peak Shank Velocity L (degrees/s) [Mean]. The symptoms of PD could be objectively quantified. By utilizing suitable motor features to construct ML models, it became possible to intelligently identify whether patients with PD were in the “ON” or “OFF” status.

The clinical diagnosis of PD is mainly based on motor symptoms, including muscular rigidity, rest tremor, bradykinesia, and postural impairment. As the duration of levodopa treatment in patients with PD increases, the duration and stability of symptom benefits decrease. As a result, the wake time is divided into the time of reduction in PD symptoms and improvement in functional status, that is, the time when levodopa provides favorable benefits (the “ON” status), and the time of PD symptom re-onset and functional status decline, that is, the time when levodopa does not provide good benefits (the “OFF” status) (25, 29). Transitions between the “ON” and “OFF” status are referred to as motor fluctuations. Motor fluctuations may be an important cause of later disability in patients with PD (30). In addition to the decline in physical function, mood swings and adverse psychological problems in patients with PD are often found in patients with motor fluctuations (31). The MDS-UPDRS-Part III is the main diagnostic tool used in clinical diagnostics and research. However, the results of many studies show the MDS-UPDRS-Part III scores are always not satisfactory in diagnosing PD because they may also lead to a certain degree of misdiagnosis (32). In addition, the evaluation process is time-consuming and heavy, which increases the burden on patients and doctors, and it is reported that many patients with PD have no opportunity to consult with a PD specialist or neurologist for professional clinical assessment, which could lead to rapid progression and disease-related complications (33).

Therefore, doctors and researchers have a strong demand for more objective and continuous evaluation and monitoring methods. With the rise of new sensor-based wearable technology, the traditional diagnosis and treatment model is changing to achieve a more objective diagnostic assessment. The WSD refers to portable and mobile devices worn on the body or embedded in clothing, such as smart glasses, watches, clothes, and pressure shoes, which contain hardware and software technologies and have special functions for collecting spatiotemporal kinematic parameters, data processing, transmission, and storage. At present, the WSD used in the field of PD can realize quantitative evaluation of PD through specific motor tasks and establish data models so that doctors can accurately analyze the movement status of patients (5, 14, 34). Compared with the results of MDS-UPDRS-Part III evaluated by doctors, WSDs were reported as a method with higher objectivity, accuracy, and sensitivity for evaluating the status of patients' motor abilities, such as bradykinesia, dyskinesia, tremor, and freezing of gait (5, 15). Therefore, by using WSDs to collect motor data from patients with PD, the internal relationship between motor data and MDS-UPDRS-Part III scores was studied, and finally, the purpose of quantifying the MDS-UPDRS-Part III scores of patients with PD was achieved. It is very important for the diagnosis and treatment of PD.

In this study, the Gait: RoM Shank L (degrees) [Mean] might be the most important motor feature. It refers to the flexion and extension range of motion of the knee joint (sagittal plane). Both the patients with PD and healthy elderly people could show reduced RoM of the shank. The reduction in RoM of the shank was more pronounced in patients with PD, and it was significantly associated with PD progression (16, 17). It had also been suggested that the RoM of the lower limb joint in patients with PD was severely reduced, and at the same time, the hip and knee significantly moved in the direction of flexion (16). Compared with healthy people individuals, the patients with PD not only had more severely reduced RoM of the lower limb joint but also showed more lower limb flexion movements throughout the gait cycle. Biomechanically, this might be to counteract the enhanced trunk flexion (16). The Gait: Stride Length L (%stature) [Mean], Gait: Stride Length R (%stature) [Mean], Gait: Peak Shank Velocity R (degrees/s) [Mean], and Gait: Peak Shank Velocity L (degrees/s) [Mean] bilaterally described the stride length and peak velocity of the shank. Both stride length and peak velocity of the shank were significantly larger in the “ON” status than in the “OFF” status. Previous studies have argued that variability analysis might be more sensitive in distinguishing gait disorders than other motor features, such as step length and step speed (18, 35, 36). In our study, however, the RoM of the lower limb joint, stride length, and peak velocity of the shank could clearly distinguish the status of patients with PD. However, in our study, the Gait: Peak Horiz. Trunk Velocity (degrees/s) [Mean] and Turn: Peak Velocity (degrees/s) [Mean] were less important than the lower limb motor features, the turn-related motor features were considered the most important factors to distinguish between patients with PD and healthy elderly people (36, 37). Similar to previous studies, patients with PD turned more slowly into the “OFF” status. Turning was a complex act that required everyone to change direction while keeping the trunk stable (37). The patients with PD seemed to have more difficulty turning because they could not precisely control the RoM of the lower limb joint, stride length, and peak velocity of the shank (37). In addition, previous studies suggested that the efficacy of levodopa in improving trunk-related symptoms in patients with PD was limited (38, 39). However, we found that the values of the Gait: Peak Horiz. Trunk Velocity (degrees/s) [Mean] and Turn: Peak Velocity (degrees/s) [Mean] were significantly higher in the “ON” status than in the “OFF” status (*p* = 1.52e-13; *p* = 4.83e-14). Furthermore, the improvement rates of two motor features were significantly and positively correlated with the ADL improvement rate of patients (R = 0.4, *p* = 6.1e−05; R = 0.27, *p* = 0.0085). This might indicate a significant effect of levodopa on improving trunk-related motor features in patients. A reduction in the RoM of arm swing was also an important motor feature for patients with PD (40). Similarly, in our study, the Gait: RoM Arm R (degrees) [Mean] was significantly smaller in the “OFF” status than in the “ON” status. However, in the correlation analysis, we did not find a significant positive correlation between the ADL improvement rate and the improvement rate of the Gait: RoM Arm R (degrees) [Mean]. It might be because walking was an important factor affecting the quality of life of patients with PD and occupied most of the exercise time in daily life (41, 42).

With their inspiring classification performances, the ML and DL algorithms have been used for constructing models for classifying between patients with PD and healthy elderly people (43–46). However, the interpretability of the model and the importance of the features incorporated into the model were ambiguous. This is the first study to develop an interpretable ML model to classify the “OFF” and “ON” status of patients with PD based on the motor features of different body parts. Our study showed that the use of more sensors in the hospital or laboratory to cover the trunk and limbs of the body more comprehensively reflects the patient's mobility, balance, walking ability, and fall risk. However, this would increase the burden and discomfort of patients and is not conducive to the development of daily monitoring of patients with PD. Therefore, the current research is moving toward a minimum number of sensors worn and daily monitoring (41, 47). In the previous studies, the optimal locations for WSDs, including on the lower limbs, trunk (waist), and upper limbs, have been discussed (41, 48–55). According to published systematic reviews, the lower limbs might be the most common location (41, 48–51). Some studies also suggested that the waist and upper limbs were the optimal locations for collecting kinematic parameters in patients with PD (41, 52–54). Peraza et al. (55) proposed an automatic gait analysis process based on DL algorithms, with data sourced from triaxial accelerometers placed on the lower limbs, trunk (waist), and upper limbs. The results showed that data from single triaxial accelerometers on the lower limbs and trunk (waist) performed better than those from the upper limbs in assessing gait in patients with PD and healthy elderly people (55). Mikos et al. (56) developed a freezing of gait detection feedback system for patients with PD, which was integrated into a single wearable device sensor attached to the ankle. The system exhibited excellent performance in classification (a sensitivity of 95.6% and a specificity of 90.2%) (56). Recently, Chen et al. (41) proposed a method for patients with PD based on an optimized interpretable DL architecture. They fixed five sensors on the lower limbs, trunk (waist), and upper limbs, to collect the motor data from patients with PD and healthy elderly people during a 10 m walking test (41). After analyzing the raw data and constructing a convolutional neural network classification model, they found that the data collected by the sensor at the waist performed best in classifying patients with PD and healthy elderly people (41). We not only constructed a classification model but also quantified the importance of motion features. In general, the importance of motor features in the lower limbs might be the highest compared to other parts of the body, which provided an evaluation method for the further selection of the best single sensor wearing position. We also found a significant effect of levodopa on improving trunk-related motor features in patients with PD, and this seemed to be contrary to previous research findings. All of these might become new directions for study in the field of Parkinson's disease and WSDs.

Some limitations are found. First, this was a retrospective study, and the absence of some data might have caused some bias in the study results. Second, all data were based on patients with PD from China, and the applicability of the NB model to other ethnic groups is unclear. Third, external verification was lacking. Therefore, the study results should be interpreted cautiously. Finally, our study did not compare patients with PD and healthy elderly people. However, contrasting the “ON” and “OFF” status of patients with PD has already demonstrated the capability of WSDs to objectively quantify the symptoms. Therefore, our study is effective.

## Conclusion

The symptoms of PD could be objectively quantified. By utilizing suitable motor features to construct the ML models, it became possible to intelligently identify whether patients with PD were either in the “ON” or “OFF” status. The variations in these motor features were significantly correlated with improvement rates in patients' quality of life. In the future, they might act as objective digital biomarkers to elucidate the changes in symptoms observed in patients with PD and might be used to assist in the diagnosis and treatment of patients with PD.

## Data availability statement

The raw data supporting the conclusions of this article will be made available by the authors, without undue reservation.

## Ethics statement

Written informed consent was obtained from the individual(s) for the publication of any potentially identifiable images or data included in this article.

## Author contributions

XW: Formal analysis, Writing – original draft. LM: Data curation, Formal analysis, Writing – original draft. PW: Writing – review & editing. YS: Writing – review & editing. PC: Conceptualization, Writing – review & editing. KW: Conceptualization, Methodology, Writing – review & editing. GZ: Writing – review & editing, Conceptualization.

## Supplementary material

Supplementary Figure S1 A flowchart of the screening process.
