# Supplementary material for: Wearable sensor devices can automatically identify the ON-OFF status of patients with Parkinson's disease through an interpretable machine learning model
Source: Front Neurol. 2024 May 1;15:1387477. doi: 10.3389/fneur.2024.1387477 (PMC11094303; doi:10.3389/fneur.2024.1387477)
Supplement: Supplementary Figure S1 — A flowchart of the screening process. [file Data_Sheet_1.ZIP › Supplementary Tables.docx]

Supplementary Table S1 Inclusion and exclusion criteria

| Inclusion criteria |
| --- |
| Age ≥ 18 years old. |
| All participants were diagnosed as clinically established PD according to Movement Disorder Society Clinical Diagnostic Criteria for PD. |
| The patients who had not undergone Deep Brain Stimulation. |
| The patients without a clear history of head injury. |
| The gait data were collected. |
| Exclusion criteria |
| The patients with PD did not experience clear fluctuations in symptoms after medication. |
| The patients had any other neurological or psychiatric disorders, such as Parkinson's syndrome, Alzheimer's disease and depressive disorder. |
| The patients with heart disease, hepatic and renal dysfunction, angle-closure glaucoma, and orthopedic diseases affecting walking. |
| The patients with leukoencephalopathy, brain tumor and other organic lesions. |
| The patients who had undergone brain surgery or gamma knife treatment. |

PD = Parkinson's disease

Supplementary Table S2 The summary and comparation of eleven motor features values between “OFF” and “ON” status.

| Motor features | OFF | ON | p* |
| --- | --- | --- | --- |
| Gait: RoM Shank L (degrees) [Mean], Median [IQR] | 55.49 [19.01] | 67.88[10.9] | 3.11e−13 |
| Gait: Stride Length L (%stature) [Mean], Median [IQR] | 59.34 [17.94] | 71.55[11.1] | 9.76e−13 |
| Gait: Stride Length R (%stature) [Mean], Median [IQR] | 59.49[16.87] | 71.3[10.54] | 1.75e−12 |
| Gait: RoM Arm R (degrees) [Mean], Median [IQR] | 7.88[9.57] | 21.04[19.24] | 1.54e−14 |
| Gait: Peak Shank Velocity R (degrees/sec) [Mean], Median [IQR] | 283.92[98.17] | 358.94[68.12] | 1.13e−11 |
| Gait: Peak Horiz. Trunk Velocity (degrees/sec) [Mean], Median [IQR] | 16.76[9.01] | 25.97[12.43] | 1.52e−13 |
| Gait: Peak Shank Velocity L (degrees/sec) [Mean], Median [IQR] | 278.96[82.75] | 356.65[76.11] | 1.72e−12 |
| Turn: Peak Velocity (degrees/sec) [Mean], Median [IQR] | 94.09[38.1] | 133.47[43.82] | 4.83e−14 |

* Wilcoxon test; IQR, interquartile range
