# Supplementary figures and images for: Wearable sensor devices can automatically identify the ON-OFF status of patients with Parkinson's disease through an interpretable machine learning model
Source: Front Neurol. 2024 May 1;15:1387477. doi: 10.3389/fneur.2024.1387477 (PMC11094303; doi:10.3389/fneur.2024.1387477)

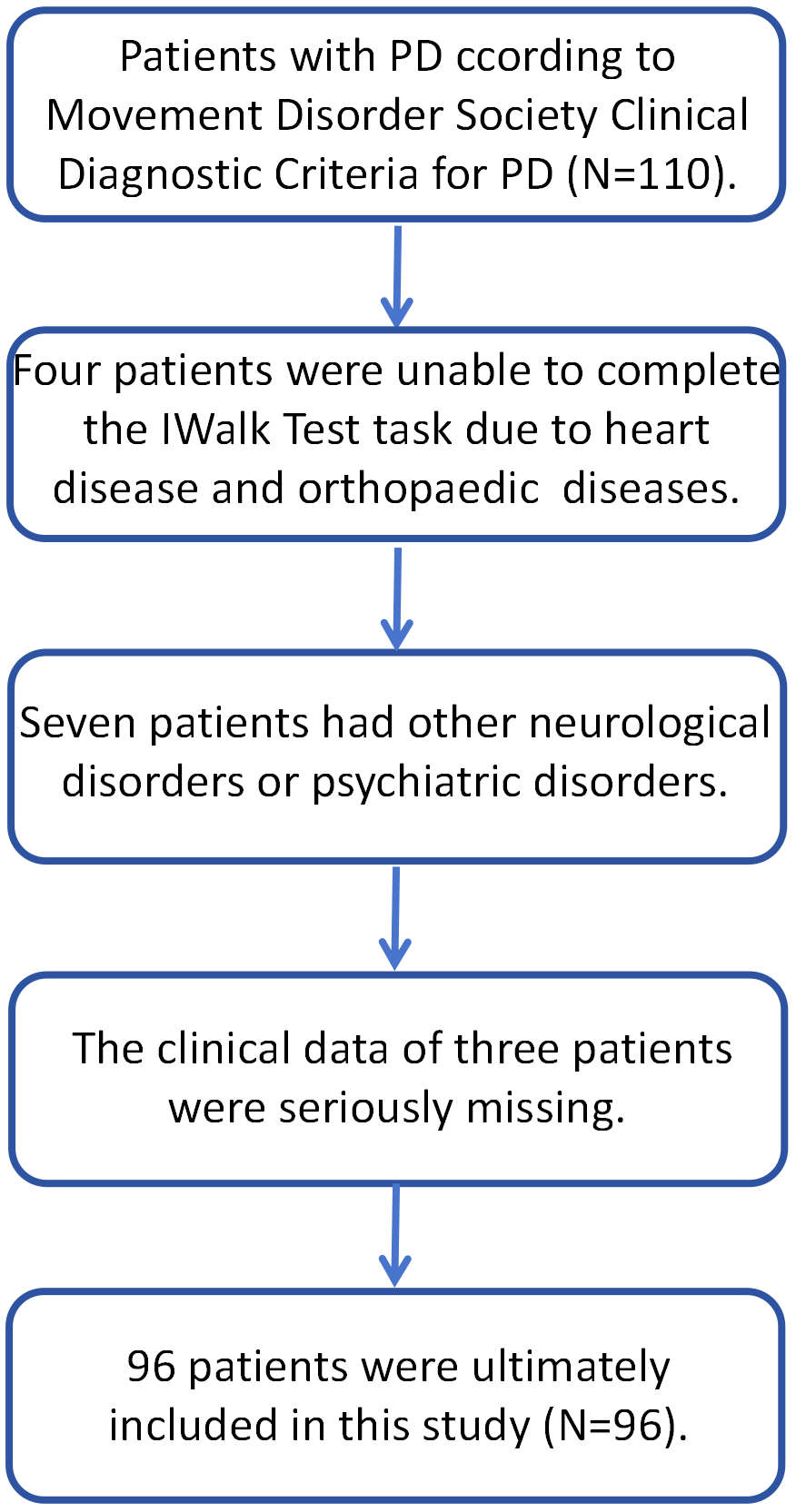

Supplement: Supplementary Figure S1 — A flowchart of the screening process. [file Data_Sheet_1.ZIP › Supplementary Figure S1.tif]
